# Listeriolysin O Membrane Damaging Activity Involves Arc Formation and Lineaction -- Implication for *Listeria monocytogenes* Escape from Phagocytic Vacuole

**DOI:** 10.1371/journal.ppat.1005597

**Published:** 2016-04-22

**Authors:** Yi Ruan, Saša Rezelj, Apolonija Bedina Zavec, Gregor Anderluh, Simon Scheuring

**Affiliations:** 1 U1006 INSERM, Université Aix-Marseille, Parc Scientifique et Technologique de Luminy, Marseille, France; 2 Department for Molecular Biology and Nanobiotechnology, National Institute of Chemistry, Ljubljana, Slovenia; University of Michigan Medical School, UNITED STATES

## Abstract

Listeriolysin-O (LLO) plays a crucial role during infection by *Listeria monocytogenes*. It enables escape of bacteria from phagocytic vacuole, which is the basis for its spread to other cells and tissues. It is not clear how LLO acts at phagosomal membranes to allow bacterial escape. The mechanism of action of LLO remains poorly understood, probably due to unavailability of suitable experimental tools that could monitor LLO membrane disruptive activity in real time. Here, we used high-speed atomic force microscopy (HS-AFM) featuring high spatio-temporal resolution on model membranes and optical microscopy on giant unilamellar vesicles (GUVs) to investigate LLO activity. We analyze the assembly kinetics of toxin oligomers, the prepore-to-pore transition dynamics and the membrane disruption in real time. We reveal that LLO toxin efficiency and mode of action as a membrane-disrupting agent varies strongly depending on the membrane cholesterol concentration and the environmental pH. We discovered that LLO is able to form arc pores as well as damage lipid membranes as a lineactant, and this leads to large-scale membrane defects. These results altogether provide a mechanistic basis of how large-scale membrane disruption leads to release of *Listeria* from the phagocytic vacuole in the cellular context.

## Author Summary

Listeriolysin-O (LLO) plays a crucial role in *Listeria monocytogenes* infection by allowing bacteria to escape from intracellular phagosomes and cells via an unknown molecular mechanism. We used high-speed atomic force microscopy (HS-AFM) supported with giant unilamellar vesicles imaging (GUVs) to characterize the interaction and dynamics of LLO with the lipid membranes at the nano-and micro-scale. We show that LLO efficiency and mode of action as a membrane-disrupting agent is strongly dependent on membrane cholesterol content and environmental pH. LLO is able to form arc pores and damage membranes as a lineactant, which is crucial for the processive membrane disruption. The latter mechanism, a previously uncharacterized mode of action for this toxin, is strongly cholesterol dependent and may provide a novel angle of attack against listeriosis.

## Introduction

Listeriolysin-O (LLO) is *Listeria monocytogenes* powerful molecular weapon in host cell invasion, which is the first step of the disease listeriosis [1]. Following accidental ingestion of *Listeria*-contaminated food, healthy humans suffer from gastroenteritis, while immunocompromised individuals are affected in the nervous system and can suffer severe damage. *Listeria* infection is treated by antibiotics, but as the development of novel antibiotics is a serious bottleneck, an improved understanding of LLO action may provide novel angles of attack to fight against this disease.

LLO is a soluble protein of 56kDa molecular weight that belongs to the cholesterol-dependent cytolysins (CDCs) protein family. CDCs are characterized by the requirement of cholesterol for their pore forming activity and by the formation of largest known transmembrane pores that can exceed 40nm in diameter [2, 3]. LLO effectively binds to lipid membranes that contain high concentrations of cholesterol [4]. Subsequently, LLO monomers oligomerize to form assemblies and then undergo a major conformation change that allows them to penetrate the membrane and form pores. LLO is different from other CDCs in that it shows pH-dependent stability, its membrane binding is diminished and its structural integrity weakened at pH of 7.4 and higher and at temperatures above 30°C [5–7]. This allows LLO to act optimally at the lower pH within the phagosomes of the infected cells, where *Listeria* is engulfed after cell entry. Membrane insertion of LLO oligomers and permeabilization of the *Listeria*-containing vacuole enables escape of *Listeria* from the phagosome into the infected cells and spread to other tissues [1, 2, 6, 8–11]. Bacterial escape to the cytosol is accompanied by uncoupling of the pH gradient between the primary phagosome and the cytosol. It was shown that this is caused by LLO-mediated membrane permeabilization that occurs soon after the entry of bacteria into the cell [11, 12]. This delays maturation of vacuoles, prevents further acidification and allows replication of bacteria [11, 13]. Larger membrane lesions of the phagocytic vacuole finally evolve and allow escape of bacteria to the cytosol of the cell and further spreading to neighboring cells [11]. The mechanisms of phagocytic membrane disruption by LLO are, however, unknown and understanding of LLO lipid membrane damaging activity would crucially improve understanding of this most important step in the *Listeria* pathogenicity mechanism.

Cholesterol-dependence, endosomolytic pore-formation, and pH-dependence of LLO have been revealed in the last decades and recent works reported structural details of its monomers and oligomeric complexes [1, 14–17]. Studies using conventional atomic force microscopy (AFM) and electron microscopy (EM) depicted CDCs on model membranes [14, 16, 18–20], yet, the slow image acquisition speed (of one to several minutes) of conventional AFM and sample fixation in EM prohibited a detailed understanding of the dynamic action of LLO. Here, we used high-speed atomic force microscopy (HS-AFM) [21, 22], a unique tool for studying the structure and dynamics of membrane processes [23, 24], and acquired HS-AFM measurements at high spatio-temporal resolution during the entire LLO action cycle from monomer assembly on the membrane surface, LLO oligomerisation, prepore-to-pore transition and finally to the processive membrane lysis by assembly of many LLO oligomers and monomers acting at formed or existing lipid membrane defects. This unique observation was independently confirmed by experiments employing giant unilamellar vesilces (GUVs) that show reduction of number of large GUVs in the presence of LLO and membrane permeabilization to compounds much larger than the diameter of previously assumed pore architecture. A comprehensive description of LLO-induced membrane damage at different membrane cholesterol concentrations and at different environmental pH values provides a mechanistic basis for understanding *Listeria* escape from phagolysosomes.

## Materials and Methods

### Protein preparation

LLO was prepared as described in Podobnik et al [16]. The protein was aliquoted and was stored in 20mM MES, pH 5.6, 100mM NaCl at a concentration of 17*μ*M. Lysenin was prepared as described in Munguira et al. [35]

### Liposome preparation

All lipids in this study were purchased from Avanti Polar Lipids, and used without further purification: Cholesterol from ovine wool specified as 98% pure and 1,2-dioleoyl-sn-glycero-3-ethylphosphocholine (DOPC) specified as 99% pure. Briefly, lyophilized lipids were dissolved in organic solution chlorophorm:methanol 3:1 vol:vol to give a final concentration of 3mM. An aliquot was poured in a glass vial and evaporated to dryness with clean nitrogen flow. The resulting lipid film was kept under reduced pressure overnight to ensure the absence of organic solvent traces. Then, the lipid film was hydrated with Milli-Q water to give a final lipid concentration of 500*μ*M, subjecting the vials to 5 cycles of agitation of 1 min, and heating ∼70°C, well above the transition temperature of the lipid mixtures studied herein. The obtained multilamellar vesicles were sonicated for 40 minutes in order to obtain LUVs. After preparation, LUVs suspensions were stored at ∼4°C and used during maximal 10 days. During all the preparation processes, samples were protected from light to avoid unspecific oxidation.

### Supported bilayer preparation

Supported Lipid Bilayers (SLBs) were prepared by fusion of Large Unilamellar Vesicles (LUVs) on the mica support, adapted from [25]. To form the SLBs, 2*μ*L of LUVs were deposited on 1.5mm^2^ freshly cleaved mica surface, which was glued with epoxy to the quartz sample stage. After 30–40 minutes incubation in a humid chamber, sample was gently rinsed with milli-Q water and never let dry.

### HS-AFM imaging

High-speed atomic force microscopy movies were acquired using 8 *μ*m-long cantilever with nominal spring constant k = 0.15N/m and resonance frequency f = 0.6MHz in solution. Both the cantilever and the rinsed mica surface with incubated bilayer were placed into a 120*μ*L imaging buffer chamber. HS-AFM was operated in oscillating mode. Small oscillation free and set point amplitude of about 1nm and 0.9nm, respectively, were used, to achieve minimum tip-sample interaction. LLO water soluble monomers were added to a final concentration of 500nM after identification of the membrane patches on the mica surface. HS-AFM measurements were performed at room temperature. Buffer of 20mM MES, pH5.6, 100mM NaCl, 5mM MgCl_2_ was used for structural observation of LLO by HS-AFM while dynamic characterizations were carried out in 20mM MES, pH5.6, 100mM NaCl, 1mM EDTA; as we found that the presence of divalents significantly slowed or inhibited LLO action probably due to stabilisation of the lipid bilayer. HS-AFM image and data processing were performed using ImageJ software with a dedicated Plugins developed for HS-AFM [26]. All further analysis, i.e. histogram distributions were analyzed in Matlab and Origin.

### Electroformation of GUVs

Giant Unilmellar Vesicles (GUVs) were prepared by the electroformation method. Lipid stock solutions of DOPC/Cholesterol 4:1 mol:mol, DOPC/Cholesterol 9:1 mol:mol, DOPC and POPC/sphingomyelin 1:1 mol:mol (for imaging lysenin activity) were prepared in chloroform. Rhodamine DHPE was added as fluorescent probe with the final concentration of 0.5mol%. 20*μ*L of lipid stock solution was placed on the conductive ITO slide and dried under reduced pressure for 30 minutes. The sucrose solution (290mM sucrose in 1mM MES, pH5.6, for preparation of DOPC/Cholesterol GUVs and 290mM sucrose, 1mM HEPES, pH7.4 for preparation of POPC/sphingomyelin GUVs) was added to the dry lipid film in the center of the O-ring and covered with another conductive ITO slide. Electroformation was carried out inside Nanion vesicle prep pro, where AC current with an amplitude of 3V and a frequency of 5Hz was applied across the ITO slides for 3 hours (for preparation of DOPC/Cholesterol GUVs) and for preparation of POPC/Sphingomyelin GUVs an amplitude decreased from 3V to 1,6V and a frequency decreased from 5Hz to 1Hz in 5 hours. GUVs were sedimented with the glucose solution (290mM glucose in 1mM MES, pH5.6 for preparation of DOPC/Cholesterol GUVs and 290 mM glucose, 1mM HEPES, pH7.4 for preparation of POPC/ sphingomyelin GUVs). The buffer was then exchanged by gentle pipetting with 20mM MES, pH5.6, 150mM NaCl for preparation of DOPC/Cholesterol GUVs and with 20mM HEPES, 150mM NaCl, pH7.4 for preparation of POPC/ sphingomyelin GUVs). All solutions used for electroformation, sedimentation and analysis with proteins were isoosmolar, the solution osmolarity was adjusted using an osmometer.

Sedimented GUVs were used immediately. They were stored at ∼4°C and never used after 4 days. During the preparation process and storage the samples were protected from light.

### Flow cytometry

The GUV suspension was mixed with LLO, dissolved in buffer (20mM MES, pH 5.6, 150mM NaCl) to final LLO concentrations of 1, 10, 100 and 500nM. The buffer was used instead of LLO solution in the negative control sample. Samples were incubated for 30 minutes at room temperature and then analyzed by flow cytometry. Flow cytometric data acquisition and analysis were performed by the PARTEC CyFlow flow cytometer with a 488nm laser and equipped with FloMax software. The presence of particles was determined by forward and side scatter (FSC/SSC) parameters, set at logarithmic gain. Minimum threshold of 70 was set at the FSC parameter to limit the measurement of the smallest vesicles and micelles. At least 15000 events were recorded for each sample analysis. Size-calibrated fluorescent beads of 1*μ*m, 3.1*μ*m, and 10*μ*m size were used to determine the appropriate size of vesicles in the sample. Flow Jo software was used for the analysis of the results.

### Imaging with confocal fluorescence microscopy

For the LLO activity experiments, GUVs suspension was mixed with buffer (20mM MES, pH5.6, 150mM NaCl) and fluorescent dextrans (FDs) and incubated at room temperature. In parallel, for the experiments with lysenin, GUVs suspension (POPC: Sphingomyelin 1:1 mol:mol) was mixed with buffer (20mM HEPES, pH7.4, 150mM NaCl) and fluorescent dextrans (FDs) and incubated at room temperature. The dextrans were passed across a gel filtration column to determine homogeneity. Fractions were collected and analyzed for permeability of GUVs and size with dynamic light scattering. Buffer alone was used instead of LLO or lysenin solution for negative control. The final concentrations were 500nM for LLO or lysenin and 1mg/ml for FDs of 4, 20, 70, 150, 2000kDa in size. Images were recorded on a Leica TCS SP5 laser-scanning microscope with a 40 × oil-immersion objective (numerical aperture = 1.25). FDs were excited at 488nm and fluorescence emission detected from 497 to 534nm. Rhodamine in the GUV membranes was excited at 543nm and fluorescence emission was detected from 573 to 604nm.

## Results

Controlled HS-AFM movie acquisition at sub-second temporal resolution of LLO assembly on model membranes allowed analyzing its action in a series of well-defined experimental conditions, notably when the protein was exposed to bilayers containing varying amounts of cholesterol (along with DOPC) and as a function of environmental pH.

DOPC/Cholesterol model membranes were formed on freshly cleaved mica HS-AFM supports by incubation of large unilamellar vesicle (LUV) suspension at room temperature (see Materials and Methods). HS-AFM allowed the direct visualization of bilayer formation from LUV adsorption to the mica surface (S1 Video). This direct imaging of membrane formation ascertains ideal placement of the subsequent HS-AFM observation of LLO action.

### Structural analysis of LLO oligomers

**Fig 1:**
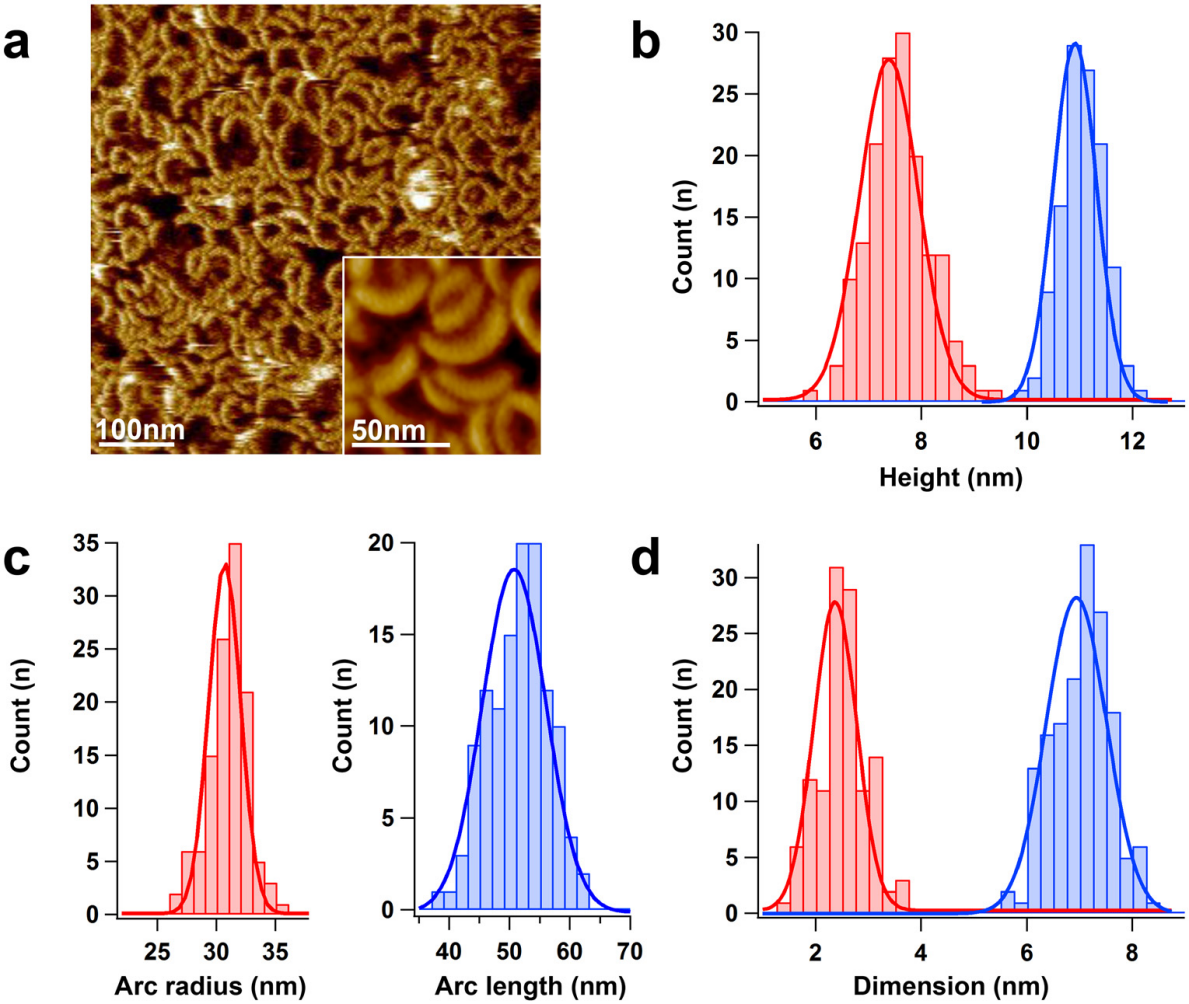
Structural characterization of LLO assemblies by HS-AFM. a) HS-AFM topography image of LLO oligomers on a DOPC:Cholesterol 4:1 mol:mol membrane observed in 20mM MES, pH5.6, 100mM NaCl, 5mM MgCl_2_, with 500nM LLO. The LLO forms arc-shaped assemblies. Inset: LLO arcs imaged at high resolution (time-averaged over 3 consecutive frames) b) Height histogram distributions of pre-pore (red) and pore (blue) complexes. The Gaussian fits indicate heights of 11.1±0.5nm (n = 120), and 7.3±0.2nm (n = 160) for the pre-pore and pore oligomers, respectively. c) The oligomers form assemblies with rather well preserved arc radius (red) of 31±3nm (n = 120) and arc length (blue) of 51±6nm (n = 120). d) The individual LLO subunits displayed dimensions of a minor axis along the arc (red) of 2.5±0.2nm (n = 120) and major axis across the arc (blue) of 7.0±0.3nm (n = 160).

LLO was added to 500nM final concentration onto the bilayers (DOPC:Cholesterol 4:1 mol:mol, at pH5.6). In typical HS-AFM image frames, the darkest image areas correspond to the membrane surface and the arc-shaped brighter structures are the protruding LLO complexes (Fig 1a). From such images the protrusion height from the membrane of the oligomeric protein complexes could be analyzed. The highest protruding clusters displayed heights of 11.1±0.5nm (Peak±FWHM) (Fig 1b, **blue**), very similar to the height of water-soluble LLO [14], and were therefore assigned to the prepore state. Later, arc-shaped complexes protruded less, only 7.3±0.2nm (Peak±FWHM), from the membrane surface, and were consequently assigned to the pore state (Fig 1b, **red**), as the height difference suggested the vertical collapse of CDCs accompanying membrane insertion [16, 19]. These results are in good agreement with previous studies characterizing the soluble and membrane structure of LLO with various techniques [6, 14, 16].

Recently the soluble state atomic-level-structure of LLO has been solved [14]. However, little is known about the detailed structure of the membrane-embedded protein complex, and studies diverge on the apparently simple question whether the LLO oligomers formed rings or incomplete arc-shaped pore structures [1, 2, 14–16, 27–29]. A recent structural model, based on X-ray crystallography and electron microscopy proposed a ring with about 50nm diameter composed of 36 LLO monomers [14]. Our HS-AFM data permits to obtain detailed insights into the characteristics of LLO pore assemblies in a native-like environment (Fig 1a). Interestingly, we never observed ring-shaped LLO assemblies. In contrast, LLO formed arcs independent on the protein density. Arcs displayed rather well-conserved structural characteristics with an average arc-length of 51±6nm (Peak+FWHM) (Fig 1c, **blue**), and an arc curvature radius of 31±3nm (Peak±FWHM) (Fig 1c, **red**). Individual LLO monomers in these arcs had dimensions of 2.5±0.2nm (Peak±FWHM) times 7.0±0.3nm (Peak±FWHM), along the arc direction and across the assembly, respectively (Fig 1d), meaning that the average arc is constituted of about 20 monomers. This implies that the final assemblies, if they would form a ring, would have a diameter of between 60 and 65nm, larger than earlier estimates [14]. However, we propose that the LLO assembly should not be regarded in that way, as we have evidence that membrane-asssociated LLO exist mainly in the arcs. We show (i) that > 95% of all assemblies are arc-shaped with rather constant dimensions, (ii) that arc radius analysis indicates a much larger effective diameter than what was proposed rendering the formation of a closed circular assembly difficult, (iii) strong evidence for the spatio-temporal separation of assembly and membrane insertion (see below) hence hampering further assembly growth once the arc transforms into the pore-state.

### Dynamics of LLO assembly on the membrane

**Fig 2:**
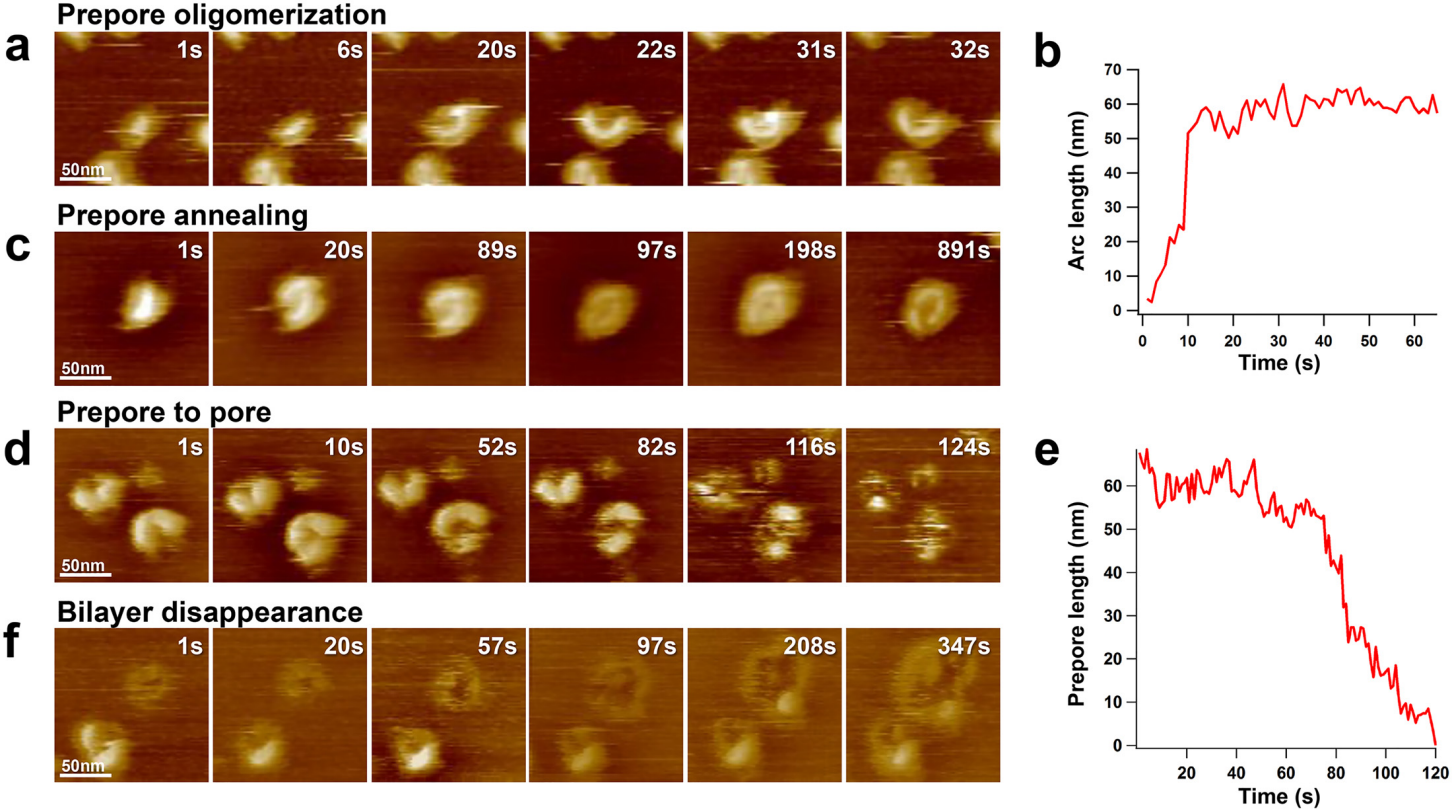
Dynamic characterization of LLO assembly and action. Buffer condition: 20mM MES, pH5.6, 100mM NaCl, 1mM EDTA, LLO final concentration 500nM. a) Prepore oligomerization process. b): The oligomerisation rate is about 5nm/s (about 2 subunits/s) until completion of an arc after about 10s, oligomerisation then stalls. c) Annealing process of neighboring arcs: arc-shaped oligomers interlock to form a final ellipsoid LLO assembly that does not further evolve. d) Membrane insertion process, i.e. prepore-to-pore transition. e) The pre-pore is stable on the bilayer for 60s and then inserts rapidly and entirely with about 2nm/s (about 1 subunits/s). f) Following oligomerisation and prepore-to-pore transition, bilayer disruption occurs at about 600nm^2^/s (see Figs 3 and 4).

Compared to conventional AFM, HS-AFM provides the possibility to assess dynamics at time-ranges of biological relevance allowing LLO action to be analyzed in real-time (S2, S3, S4 and S5 Videos). HS-AFM directly revealed the assembly of LLO into arc-shaped oligomers on the membrane surface (Fig 2a). We reason that the membrane disturbance of a monomer penetrating into one membrane leaflet is energetically costly and that bringing several units together minimizes this energy cost, driving oligomerization. This process occurred on time scales as short as 10s at 500nM LLO concentration. Once arcs have reached maturity, i.e. assembling about 20 monomers in an arc of about 50nm length, assembly stalled and further length analysis over 60s revealed length fluctuations of a few nanometers, the size of a single subunit and within the measurement error (Fig 2b). The formation of complete circular ring-shaped complexes from arc-shaped lysteriolysin was never observed. Closest to this, annealing of neighboring arcs was regularly observed. In this case, arc-shaped oligomers were interlocked to exhibit a rather ellipsoidal surface contour that did not further evolve as a function of time (Fig 2c). Membrane insertion has only been observed when rather advanced arc assemblies have been formed, indication that the prepore-to-pore transition needs an advanced state of oligomerization and might therefore be cooperative (Fig 2d). Quantitatively, single molecule analysis showed varying residence lifetime of prepore complexes on the membrane of up to minutes, followed by insertion that is completed within seconds. This more rapid membrane insertion compared to the residence lifetime of prepores is further evidence for a cooperative conformational change within the units of the prepore arcs for membrane insertion (Fig 2e). Further oligomerisation of already membrane embedded arc-shaped oligomers with other existing LLO arcs in the insertion state was never observed. These results show that the oligomerisation and membrane insertion actions of LLO are spatiotemporally uncoupled. Once LLO is in the membrane, the dynamic destruction of the bilayer could directly be observed: membrane defects occur inside the arc and grow by lateral propagation of the toxins in the membrane (Fig 2f). It is notable that the perimeter edged by LLO remaining in the prepore state does not propagate in membrane disruption (Fig 2f, S5 Video)

### Membrane destruction by LLO activity

While the LLO membrane destructive action in dependence of cholesterol has been described in detail [1, 4, 15, 30, 31], and recent AFM analysis described the LLO assembly structures at high resolution [17], the morphology and dynamics of LLO action on cholesterol containing membranes remains unknown. The versatility of HS-AFM to dynamically image bilayers of various compositions and under varying buffer conditions allows structural description of the mode of action of LLO in detail. Notably, the influences of cholesterol and pH on LLO action have been studied, both essential factors during the cell infection process.

### LLO activity depends on membrane cholesterol content

As earlier reported, LLO activity is optimized at slightly acidic pH [5, 6]. We therefore carried out measurements to analyze how varying concentrations of membrane cholesterol influences LLO action in a buffer at pH5.6, containing 20mM MES, 100mM NaCl and 1mM EDTA. These measurements were performed by adding LLO to a final concentration of 500nM onto DOPC/Cholesterol model membranes with varying cholesterol content. Our results demonstrate a novel dynamic view of LLO membrane activity. Notably, two types of membrane destruction, either from the inside of the membrane, following membrane insertion, or from membrane borders could be distinguished. In the first case, the pre-pore to pore transition is indispensible, while in the second case lineactivity alone is sufficient (S6, S7, S8, S9 Videos). We conceptualize the lineactant activity as the 2D (two-dimensional) analogue to surfactant activity in 3D. A lineactant is a line-tension modifying agent that ‘solubilizes’ the 2D membrane.

**Fig 3:**
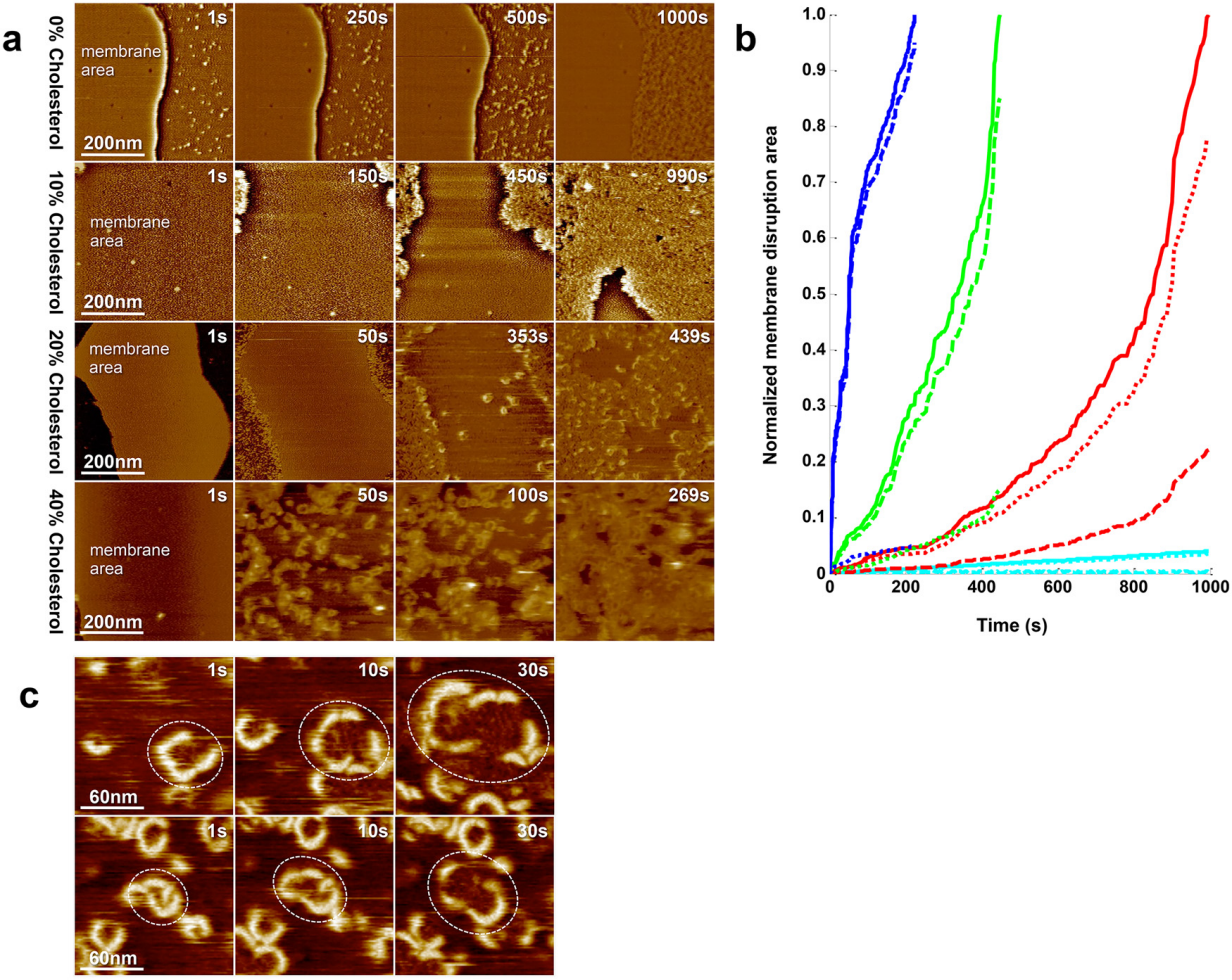
LLO activity depends on membrane cholesterol content. a) HS-AFM frames of the time-course of LLO membrane disruption as function of different membrane cholesterol concentrations: from top to bottom: 0mol%, 10mol%, 20mol%, 40mol% cholesterol. All measurements were carried out in 20mM MES, pH5.6, 100mM NaCl, 1mM EDTA, LLO final concentration 500nM. At 0mol% cholesterol, LLO monomers only adsorb to mica, and no LLO membrane disruption is observed. At 10mol% cholesterol, LLO acts from membrane borders, and no typical oligomer structures are observed on the membrane. At 20mol% cholesterol, LLO pore formation on the membrane is detected, yet the major membrane disruptive activity occurs from the membrane border. At 40mol% cholesterol, typical LLO oligomers are formed on the membrane and lead to membrane destruction. b) Normalized membrane disruption area versus observation lag-time at varying membrane cholesterol content. Quantitatively, ∼0nm^2^/s, ∼300nm^2^/s, ∼600nm^2^/s, and ∼1200nm^2^/s membrane disruption velocity are detected at 0mol% (cyan), 10mol% (red), 20mol% (green), 40mol% (blue) membrane cholesterol concentration, respectively. The hyphen line is the membrane disruption velocity from oligomer membrane insertion, while the dashed line documents membrane disruption from bilayer borders. The solid line is the total membrane disruption velocity. c) Detailed illustration of the lateral expansion of arc pores during membrane destruction of a 40mol% cholesterol containing membrane. Destruction only progresses on defect borders decorated by LLO.

At 0mol% cholesterol, S6 Video, the membrane is basically resistant to LLO (Fig 3a, **1** *^st^* **row**). As a function of incubation time with 500nM LLO, no membrane damage is detected, not even after more than 15 minutes. LLO adsorbes next to the bilayer on the mica and is unable to adhere or damage the membrane border.

At 10mol% cholesterol, S7 Video, the membrane becomes senistive to LLO (Fig 3a, **2** *^nd^* **row**). However, under these conditions LLO was never observed to form arc-shaped assemblies on the membrane and/or insert the membrane, yet LLO could act from the membrane edges, ‘solubilizing’ the membrane from the sides as a lineactant. This shows that 10mol% cholesterol are not enough for oligomerisation and insertion, but is a neccessity for membrane disruption. After about 20 minutes, only small membrane fragments remained.

At 20mol% cholesterol, S8 Video, LLO oligomerization, arc-formation and prepore-to-pore transition were observed (Fig 3a, **3** *^rd^* **row**). LLO penetrated the membrane rapidly under these conditions. In addition we noted that LLO subsequently disrupted the membrane both from within the membrane, where the initial arc-complexes served as nucleation points for further membrane disruption, and from membrane edges.

At 40mol% cholesterol, S9 Video, LLO activity is further accelerated (Fig 3a, **4** *^th^* **row**). Arcs form rapidly everywhere on the membrane, insert and lyse the bilayer; within about 5 minutes of LLO action the entire membrane was destroyed.

Beyond morphological aspects of the membrane disruption dynamics, the HS-AFM movies allowed numerical analysis of the membrane disruption velocity (Fig 3b). The membrane area was computationaly analyzed in each image frame and the disruption process plotted as a function of time. The average velocity could be calculated as roughly 0nm^2^/s, ∼300nm^2^/s, ∼600nm^2^/s, and ∼1200nm^2^/s membrane disruption velocity for 0, 10, 20 and 40 mol% membrane cholesterol content, respectively, at constant 500nM LLO concentration. Although membrane disruption is not linear, because the circumference-area-ratio changes as a function of time-course of LLO-action (and this is particularly important for the conditions in which LLO acts mainly from the membrane edges) and the experiment is limited to relatively small observation areas, a rough linear correlation between LLO membrane disruption efficiency and membrane cholesterol-content emerges (Fig 3b). Membrane disruption only occurred from borders or defect edges that were decorated with LLO arcs. These arcs appeared to remain of constant size during dynamic large-scale membrane defect generation (Fig 3c, S9 Video). Based on the HS-AFM observations, it cannot be determined whether the retracting lipid material during the disruption process was solubized by the protein and released into solution or moved out of the membrane defect.

### LLO activity depends on environmental pH

LLO was shown to act efficiently in very different environments such as the phagolysosomal membrane, where the pH is low, as well as at the plasma membrane level, where it is exposed to the physiological pH [4, 10–12, 20]. To learn more about the pH-dependence of LLO membrane disruption, we designed our next measurements under three typical pH conditions, i.e. acidic pH5.6, neutral pH7.6, and alkaline pH9.6, on a DOPC:Cholesterol 4:1 mol:mol membrane, knowing that this cholesterol content is typically close to physiological and allows the toxin to act efficiently [32] (S10 and S11 Videos).

**Fig 4:**
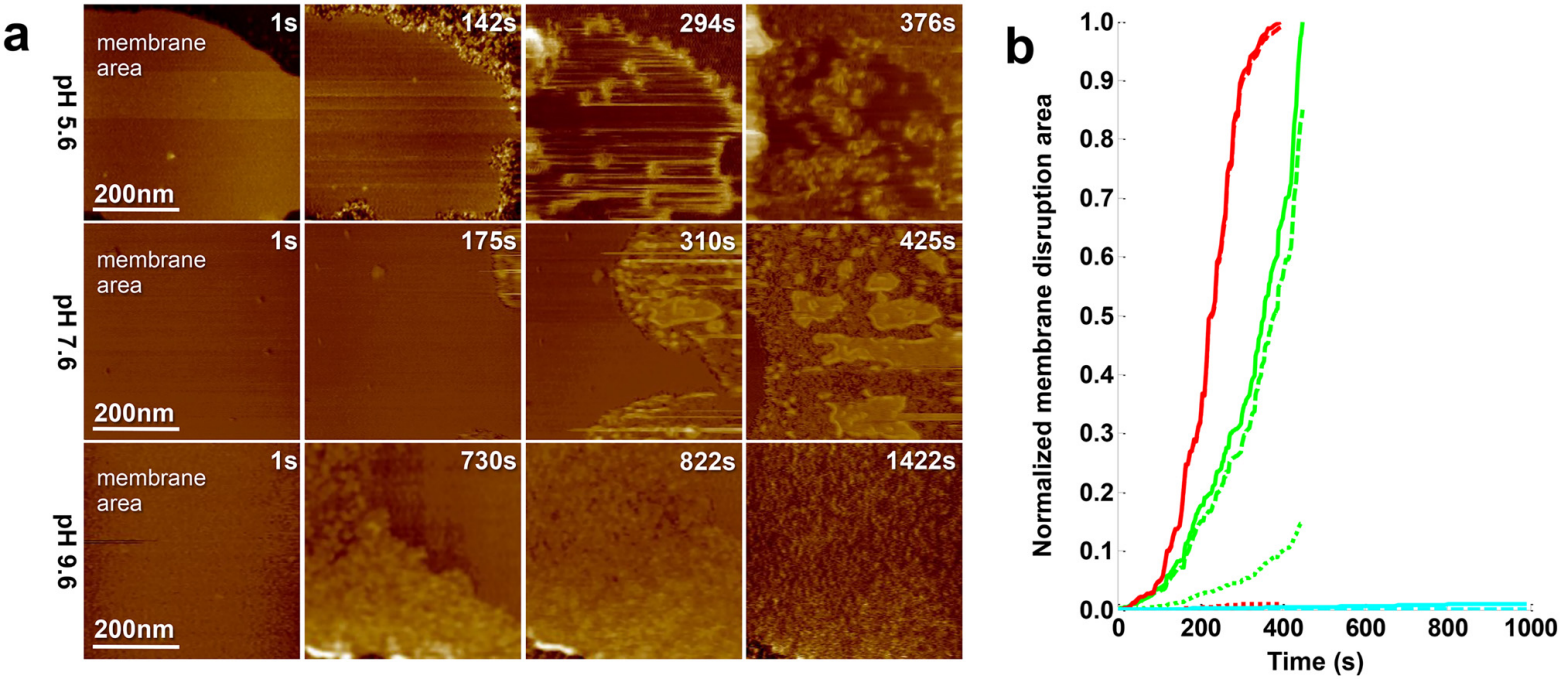
LLO activity depends on the environmental pH. a) HS-AFM frames comparing LLO activity at different environmental pH on a 20mol% cholesterol containing bilayer. All measurements were carried out in 20mM MES, 100mM NaCl, 1mM EDTA, LLO final concentration 500nM. At pH5.6 membrane disruptive activity is efficient from pores and propagating from generated defects and membrane borders. At pH7.6, the membrane is disrupted from its edges. LLO oligomers formed at membrane borders do not display typical arc-shaped architecture. At alkaline pH 9.6, the efficiency of LLO is abolished. LLO float on the membrane surface without binding or insertion. b) Normalized membrane disruption area versus observation lag-time. The membrane disruption velocity is ∼600 nm^2^/s, ∼650nm^2^/s, and ∼0nm^2^/s at pH5.6 (green), pH7.6 (red), and pH9.6 (cyan), respectively. The hyphen line is the membrane disruption speed from LLO oligomer insertion. The dashed line represents membrane disruption from the bilayer border. The solid line is the total membrane disruption.

As reported above, at pH5.6 LLO is highly efficient (Fig 4a, **1** *^st^* **row**): The entire functional path is supported under such conditions, notably, LLO undergoes prepore-to-pore transition creating novel membrane defects and then disrupts the membrane at about ∼600nm^2^/s (Fig 4b).

At pH7.6, S10 Video, membrane disruption could also be observed, but only from membrane edges and no well-defined prepore oligomers were observed on the membrane (Fig 4a, **2** *^nd^* **row**). Nevertheless, this experiment demonstrated that the inactivation of LLO at neutral pH was not complete, despite that neutral pH hampered the formation of oligomeric complexes and insertion, the membrane ‘solubilisation’ process still occurred. Importantly, membrane disruptions occurred at the same velocity as at acidic pH, ∼600nm^2^/s (Fig 4b).

In contrast, at alkaline pH9.6, S11 Video, LLO was inactive and no membrane disruption (∼0nm^2^/s) could be detected. Fast LLO diffusion along the membrane surface was detected, but no membrane attachment, oligomerisation or penetration could be observed, despite the presence of cholesterol in the membrane (Fig 4a, **3** *^rd^* **row**). This finding indicates that LLO at alkaline pH does neither engage into protein-lipid or protein-protein interactions, probably due to unfavorable charges exposed on the protein surface, in agreement with earlier work [4].

### LLO progressively permeabilizes GUVs for large compounds

**Fig 5:**
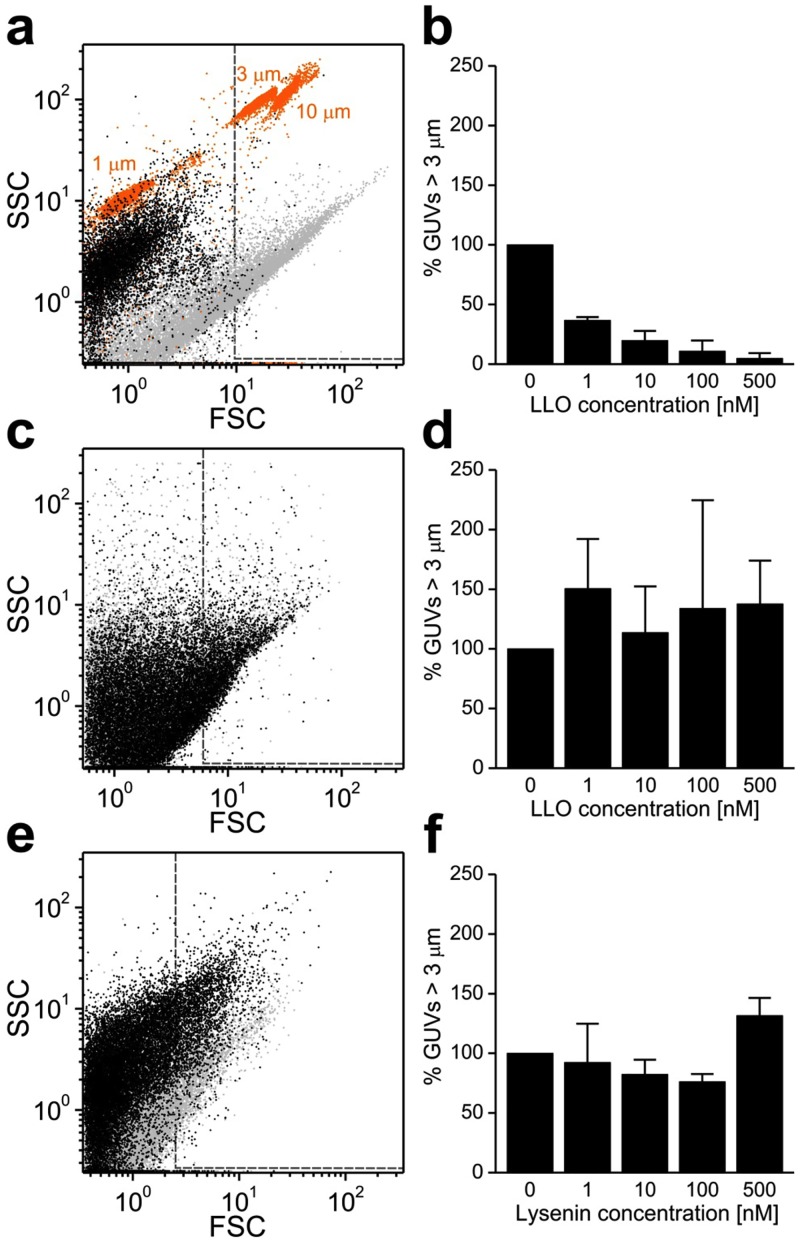
Effect of LLO on the size of giant unilamellar vesicles (GUVs). a) Flow cytometry analysis of GUVs destruction by LLO: GUVs in the absence (gray dots) and presence of 500nM LLO (black dots). Size-calibrated fluorescent beads (orange) are shown for comparison. b) Percentage of GUVs larger than 3*μ*m, on the right of the gray line in a), were quantified for different LLO concentrations. c) Flow cytometry analysis of DOPC GUVs in the absence (gray dots) and presence of LLO (black dots). d) Quantification of data presented in c) performed as in b). e) Flow cytometry analysis of POPC/sphingomyelin 1/1 (mol%) GUVs in the absence (gray dots) or presence (black dots) of lysenin. f) Quantification of data presented in e performed as in b. The data from three independent experiments are shown in panels b, d and f. Average ± S.D.

**Fig 6:**
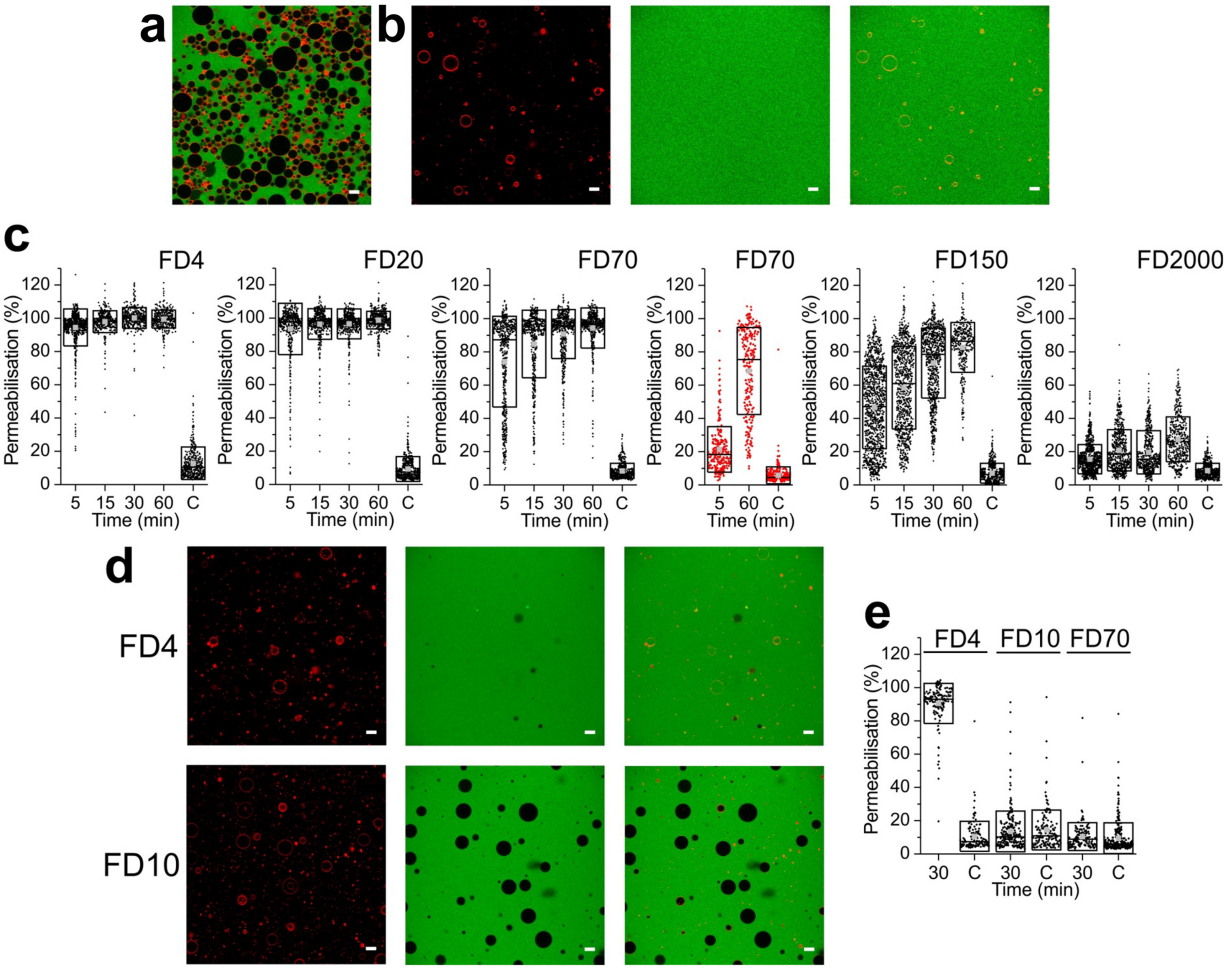
Permeabilisation of GUVs by LLO and lysenin. a) GUVs in the absence of LLO and in the presence of 1mg/ml FD4 in the outside medium. b) GUVs (left, red membrane stain) in the presence of 500nM LLO and in the presence of 1mg/ml of FD4 after 60 min incubation (middle). The right panel represents superimposed image). c) Quantification of GUVs permeabilisation in the presence of 500nM LLO and fluorescent dextrans (FD) of various sizes upon incubation at different time points. ‘C’ on the x-axis marks, control in the absence of LLO and imaged after 60 min. The data presented in red for FD70 are for membrane composition, which contains 10 mol% of cholesterol, where LLO cannot form pores effectively. d) GUVs (left, red stain) in the presence of 500nM lysenin and in the presence of 1mg/ml of FD4 or FD10 after 30 min incubation (middle). The right panel represents superimposed image). e) Quantification of GUVs permeabilisation in the presence of 500nM lysenin and fluorescent dextrans (FD) of various sizes after 30 min. Only the smallest dextran is permeable through the lysenin pore of fixed diameter of about 3.5nm.

The HS-AFM experiments have revealed that LLO can induce large-scale membrane destruction. In order to prove that these observations on supported lipid bilayers were representative for LLO action, GUVs were formed in order to independently confirm LLO membrane destructive activity. GUVs were prepared by electroformation from DOPC/Cholesterol 4:1 mol:mol lipid mixture (lipid composition used in the HS-AFM experiments) and imaged at pH5.5. GUVs size distributions were analyzed by flow cytometry in the presence of various concentrations of LLO. Flow cytometry revealed that presence of LLO caused drastic reduction in the number of GUVs that were larger than 3*μ*m in diameter (Fig 5a and 5b). At 500nM LLO concentration (concentration used in HS-AFM experiments) only few GUVs remained in the mixture (Fig 5b). Confocal microscopy confirmed these results and showed a drastic reduction in the number of GUVs in the presence of LLO, in addition to GUV permeabilization (Fig 6a–6c). We have performed several additional control experiments. The population of large GUVs composed of DOPC alone is not decreased in the presence of LLO, in agreement with the known LLO inability to associate with the membranes devoid of cholesterol (Fig 5c and 5d). As another control we have used pore forming toxin lysenin, which belongs to the aerolysin-like pore forming toxin family [36]. Lysenin forms stable *β*-barrel pores formed by 9 subunits with a pore diameter of approximately 3.5nm, which are significantly smaller than pores of CDCs [35, 37]. Lysenin does not show lineactant activity on lipid membranes as imaged by HS-AFM [35, 38]. In agreement with these literature data, the population of large vesicles does not decrease in number upon addition of comparable lysenin concentration ranges (Fig 5e and 5f).

Furthermore, HS-AFM results showed that in the presence of cholesterol LLO arc formation is rapid and that arcs form pores (Fig 2). Most importantly, HS-AFM evidenced a novel LLO activity mechanism where membrane inserted LLO has high lineactant efficiency and successively destroys membrane on large-scale. Functional pore formation was analyzed in the GUVs system by monitoring uptake of fluorescent dextrans of various sizes. We have added fluorescent dextrans of various sizes, FDX, where X denotes the size of the dextran in kDa, to the exterior volume of the GUVs in the presence of LLO and checked for fluorescence equilibration at different time points. Dextrans of 4kDa and 20kDa dextran, with estimated diameter of 2.8nm and 6.6nm, respectively, equilibrated readily at 5 min (Fig 6c, **1** *^st^* **and 2** *^nd^* **panels**), while larger dextrans needed more time for equilibration. Particularly interesting is the 150kDa dextran, which is approximately 17nm in diameter, which poorly equilibrate initially, but almost fully after 60 minutes (Fig 6c, **5** *^th^* **panel**). This dextran is too large to equilibrate through arc pores. Finally, the huge FD2000 showed some membrane permeability after 60 minutes (Fig 6c, **6** *^th^* **panel**). We have also used DOPC membranes with 10 mol% of cholesterol. We show that 70 kDa dextran could only enter these GUVs at much later times (60 min), but not at 5 min as in the presence of 20 mol% cholesterol or more (Fig 6c, **4** *^th^* **panel, data in red**). This is in agreement with HS-AFM data where at this cholesterol concentrations LLO disrupt membranes primarily by lineactant activity but only rarely pores are formed (Fig 3a).

In order to check whether the observed effects are specific to LLO, we have used lysenin, as a negative control. As expected lysenin pores allowed passage of FD4 across the membranes, but not other dextrans, such as FD10 or FD70 (Fig 6d and 6e)[36, 37]. This experiment with a toxin of fixed pore size illustrates that LLO indeed presents a second functional mechanism that is qualitatively different from other toxins, namely in its capacity of creating large-scale membrane damages as a function of time.

These experiments on GUVs altogether confirm the HS-AFM imaging on supported membranes. While the kinetics in these experiments are somewhat slower compared to HS-AFM, the data further supports the model in which small arc pores initially formed are nucleators for large-scale membrane lesions by LLO.

## Discussion

Here we present a first dynamic analysis of LLO membrane activity at high-spatio-temporal resolution. A DOPC/Cholesterol model membrane system was used at various cholesterol content bathing in buffers of various pH. These experimental conditions combined with the capacity of dynamic imaging, allowed us to acquire a detailed understanding of the molecular action of LLO.

**Fig 7:**
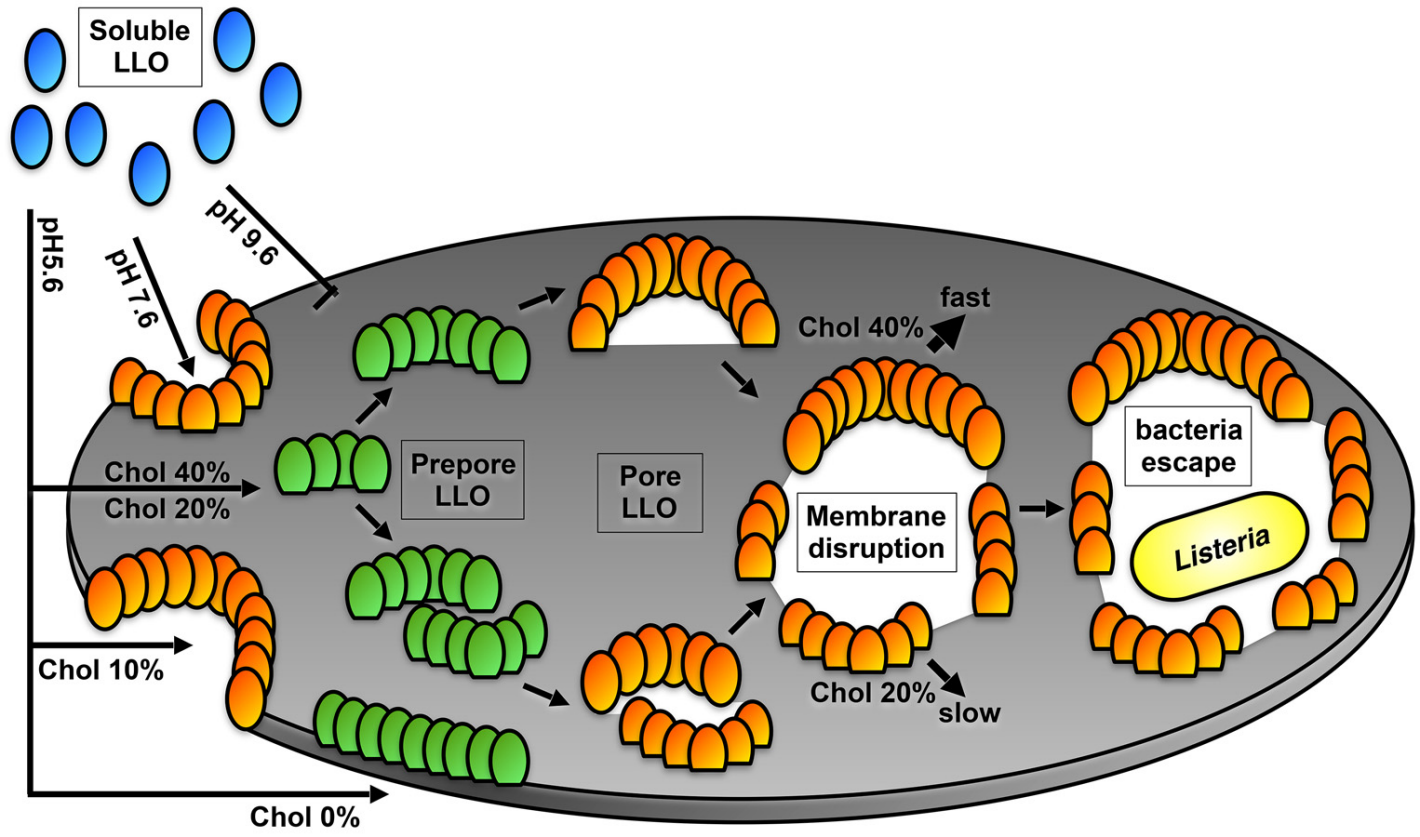
Schematic representation of LLO membrane disruptive action summarizing the presented results. LLO action depends on membrane cholesterol content and environmental pH. LLO forms arc-shaped pores and creates large-scale defects as a lineactant for *Listeria* escape. Soluble LLO is represented in blue, pre-pore LLO in green, and pore LLO in orange, respectively.

There are four key statements that characterize the function of LLO (Fig 7). First, LLO associates to the membrane: preconditions to membrane association are mildly acidic pH and the presence of at least 20mol% membrane cholesterol. Second, LLO oligomerises: preconditions for oligomerisation are also mildly acidic pH and at least 20mol% membrane cholesterol. In contrast to preconceptions and models [14, 16], LLO does not oligomerize in full circles in the used membrane lipid composition (DOPC/cholesterol). LLO forms arc-shaped assemblies of about 50nm in length and a curvature radius of about 30nm comprising about 20 subunits. Third, the prepore-to-pore transition leads to membrane insertion: this process is rapid, and needs slightly acidic pH, as reported before [1, 4–6, 10, 16]. Its efficiency is favored by cholesterol-content, maybe because cholesterol intercalates between lipids and hence diminishes lipid-lipid interactions. In this context it is notable that some CDCs, e.g. ILY, bind protein receptors, but still need cholesterol for insertion [33]. The prepore-to-pore transition seems to comprise a cooperative aspect, as insertion of monomers or small oligomers were not observed, and as the insertion of arcs was faster than the residence lifetime of the same arcs before the first subunits inserted. Importantly, the oligomerisation process and the membrane insertion are uncoupled, as oligomerisation in well defined structures takes only place in the prepore state. Assemblies inserted into the membrane are sufficient to create membrane defects allowing further membrane disruption. Fourth, membrane disruption takes place: The ‘solubilization’ process of the membrane is roughly linear with membrane cholesterol content (a further indication that cholesterol intercalation between lipids favros LLO action) and scaled at 500nM LLO concentration with about (∼300nm^2^/s) at 10mol% cholesterol. This membrane lysis can start either from a pore formed by LLO, or from a membrane edge, i.e. membrane defect, in the experimental system. It is important to note that the ‘solubilization’ process works already at 10mol% cholesterol and is pH-independent, separating completely the membrane disruption process from the membrane insertion process that is pH-dependent.

We propose that these findings have importance in a physiological context of listeriolysis. In contrast to other CDCs, LLO is not only designed to make holes into the membrane to destroy ion or nutrient gradients. One of the major tasks of LLO is to allow escape of a large bacteria from the intracellular phagocytic vacuole. For this, membrane disruption (‘solubilization’) is needed [11–13]. We quantitatively reveal that LLO membrane disruption involves pore formation into a continuous membrane, and disrupting the membrane from the borders, i.e. continuing to disrupt membrane from pre-existing damages (Fig 7). While the first necessitates slightly acidic pH, the second is maintained at physiological pH. This is the first experimental distinction of these different modes of action and allows understanding that pH sensitivity is crucial at the very early stages of LLO action. At an advanced stage, when the pH in the phagocytic vacuole has leveled with the cytoplasm through ion- and proton-gradient dissipation through small LLO pores, the second mechanism is sufficent for further membrane disruption and *Listeria* release. The membrane ‘solubilizing’ action is preserved and well efficient at neutral pH, and therefore, it allows complete disruption of the vacuolar membrane. This second step is likely enhanced with *Listeria* phospholipases that were shown to act in concertion with LLO. Phospholipases hydrolytic activity on phagosomal lipid membrane may further expose hydrophobic parts of the membrane, where LLO could act upon [34, 39]. The membrane ‘solubilisation’ proceeds through activity of LLO at the membrane exposed sites in the pore formed by inserted arcs and is mechanistically a lineactant process, as LLO were permanently imaged at the processive borders progressing in membrane destruction. Altogether, experiments presented here provide a solid basis for an understanding of the dynamics of membrane damage induced by LLO in listeriolysis.

## Supporting Information

S1 Video Supplementary Movie 1 Bilayer Formation. Direct visualization of supported lipid bilayer formation from LUV adsorption to the mica surface. Movie parameters: Image size: 600nm. Full color scale: 5nm. Image acquisition speed: 1s.
(MOV)

S2 Video Supplementary Movie 2.1 Prepore Oligomerisation. Direct visualization of the assembly of LLO into an arc-shaped oligomer complex on the membrane surface. Movie parameters: Image size: 150nm. Full color scale: 13nm. Image acquisition speed: 1s.
(MOV)

S3 Video Supplementary Movie 2.2 Prepore Oligomerisation And Annealing. Direct visualization of the formation and annealing process of arc-shaped LLO complexes into an elliptical ensemble. Movie parameters: Image size: 150nm. Full color scale: 13nm. Image acquisition speed: 1s.
(MOV)

S4 Video Supplementary Movie 2.3 Prepore To Pore Transition. Direct visualization of the prepore-to-pore transition of LLO arc-shaped complexes. Movie parameters: Image size: 150nm. Full color scale: 13nm. Image acquisition speed: 1s.
(MOV)

S5 Video Supplementary Movie 2.4 Membrane Disruption. Direct visualization of the dynamics of LLO-mediated bilayer destruction. Movie parameters: Image size: 150nm. Full color scale: 13nm. Image acquisition speed: 1s.
(MOV)

S6 Video Supplementary Movie 3.1 pH5.6 0mol%chol. In absence of cholesterol, the membrane is resistant to LLO. No observation of membrane disruption at 0mol% cholesterol content in buffer at pH5.6 and 500nM LLO concentration. Movie parameters: Image size: 600nm. Full color scale: 7nm. Image acquisition speed: 3s.
(MOV)

S7 Video Supplementary Movie 3.2 pH5.6 10mol%chol. Direct visualization of membrane disruption at 10mol% cholesterol content with 300nm^2^/s velocity, at pH5.6, LLO concentration 500nM. Movie parameters: Image size: 600nm. Full color scale: 13nm. Image acquisition speed: 3s.
(MOV)

S8 Video Supplementary Movie 3.3 pH5.6 20mol%chol. Direct visualization of membrane disruption at 20mol% cholesterol content with 600nm^2^/s, at pH5.6, LLO concentration 500nM. Movie parameters: Image size: 600nm. Full color scale: 13nm. Image acquisition speed: 3s.
(MOV)

S9 Video Supplementary Movie 3.4 pH5.6 40mol%chol. Direct visualization of membrane disruption at 40mol% cholesterol content with 1200nm^2^/s, at pH5.6, LLO concentration 500nM. Movie parameters: Image size: 600nm. Full color scale: 13nm. Image acquisition speed: 3s.
(MOV)

S10 Video Supplementary Movie 4.1 pH7.6 20mol%chol. Direct visualization of membrane disruption at pH7.6 with 600nm^2^/s, LLO concentration 500nM. Movie parameters: Image size: 600nm. Full color scale: 13nm. Image acquisition speed: 3s.
(MOV)

S11 Video Supplementary Movie 4.2 pH9.6 20mol%chol. Direct visualization of membrane disruption at pH9.6 with 600nm^2^/s, LLO concentration 500nM. Movie parameters: Image size: 600nm. Full color scale: 13nm. Image acquisition speed: 3s.
(MOV)
